# Bony metastases following complete resection of periosteal chondrosarcoma

**DOI:** 10.1186/s12957-015-0545-2

**Published:** 2015-03-26

**Authors:** Xiwei Liu, Li Min, Ganjun Chen, Song Hong, Chongqi Tu

**Affiliations:** Department of Orthopedics, West China Hospital, Sichuan University, Guoxue xiang37#, Chengdu, 610041 China; Department of Pathology, West China Hospital, Sichuan University, Guoxue xiang37#, Chengdu, 610041 China

**Keywords:** Fibula, Periosteal chondrosarcoma, Multiple bone metastases, Cortical invasion, MRI, PET/CT

## Abstract

Periosteal chondrosarcoma (PC) is a rare low-grade malignant cartilaginous tumor originating on the bone surface. Wide surgical resection is the recommended treatment. Prognosis is usually good if surgery is adequate. Metastasis is late and very rare. We present the clinical, radiographic, and pathological features of a PC accompanied with fibular cortical invasion in a 30-year-old woman. Wide resection was performed at presentation, but a whole-body positron emission tomography/computed tomography (PET/CT) examination 10 months after operation showed multiple bone metastases (MBM) especially in the spine, pelvis, bilateral femurs, and humeri without local recurrence. To the best of our knowledge, the present report is the first concerning a PC with so extensive postoperative MBM but without local recurrence.

## Background

PC is a rare low-grade malignant cartilaginous tumor originating on the bone surface, accounting for less than 2% of all chondrosarcomas and about 0.2% of all bone tumors [1-3]. Wide surgical resection is the recommended treatment. Prognosis is usually good if surgery is adequate. Metastasis is late and very rare [4].

We report a grade 2 PC with cortical invasion in the left proximal fibula of a 30-year-old woman. MBM happened especially in the spine, pelvis, bilateral femurs, and humeri in the absence of local recurrence at 10 months after operation.

## Case presentation

A 30-year-old woman presented with a 1-year history of a gradually enlarging surface lesion on the posterolateral aspect of left proximal shank. She denied the history of infection, major traumatic injury, and familial history of cancer. Physical examination revealed a hard, painless, motionless swelling on the posterolateral aspect of the left proximal fibula. The overlying skin and local temperature was normal. The left lower extremity demonstrated full range of motion without neurovascular deficits. All laboratory data were within normal limits.

**Figure 1:**
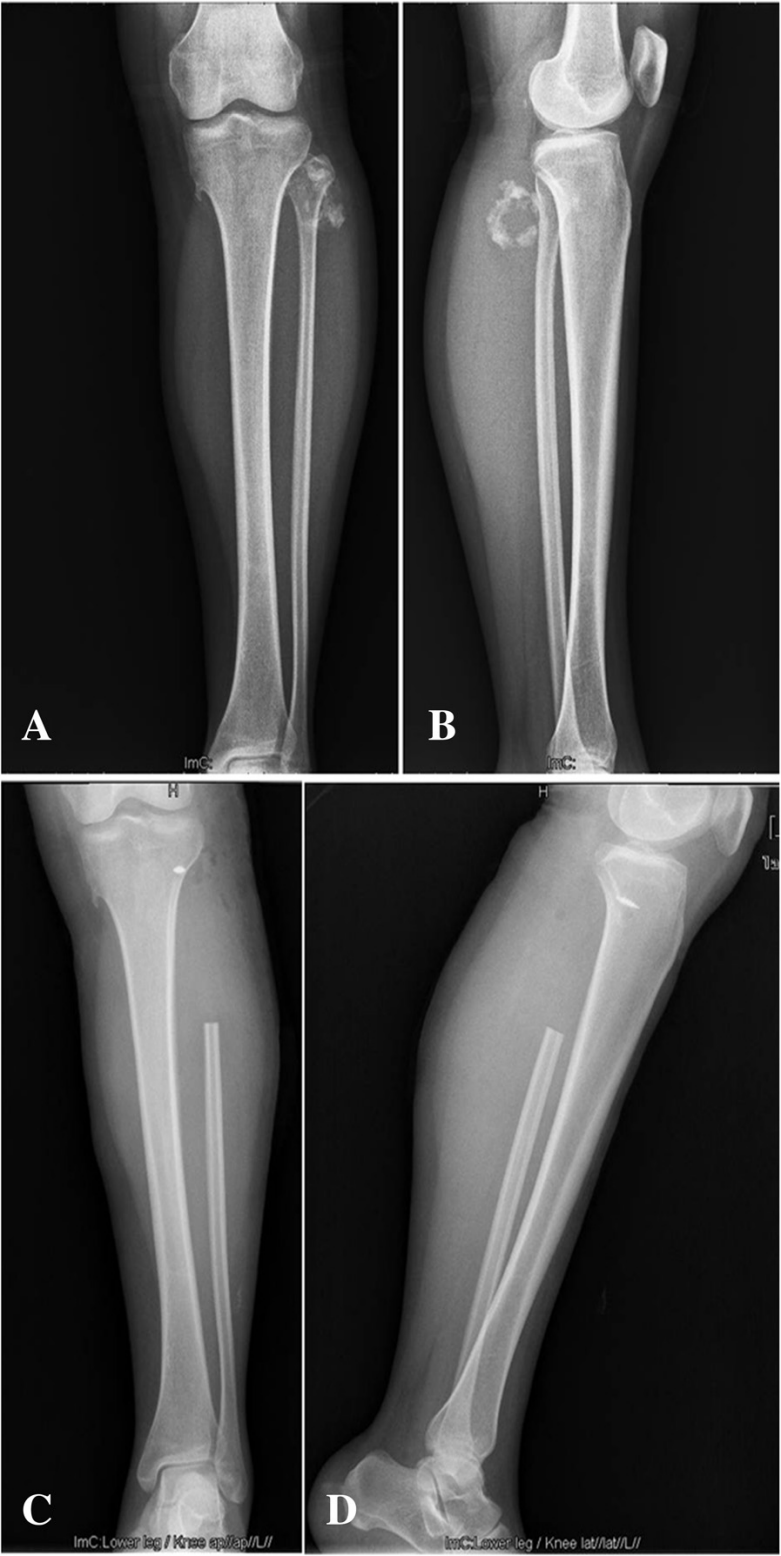
**Radiographs of the patient’s left shank. A**, **B** Anteroposterior and lateral views show a bone tumor with a ‘popcorn’ and ‘smoke ring’ pattern of calcification on the posterolateral aspect of the left proximal fibula, respectively. **C**, **D** Postoperative radiographs show the absence of the left proximal fibula and an anchor inserted the lateral tibial metaphysis.

**Figure 2:**
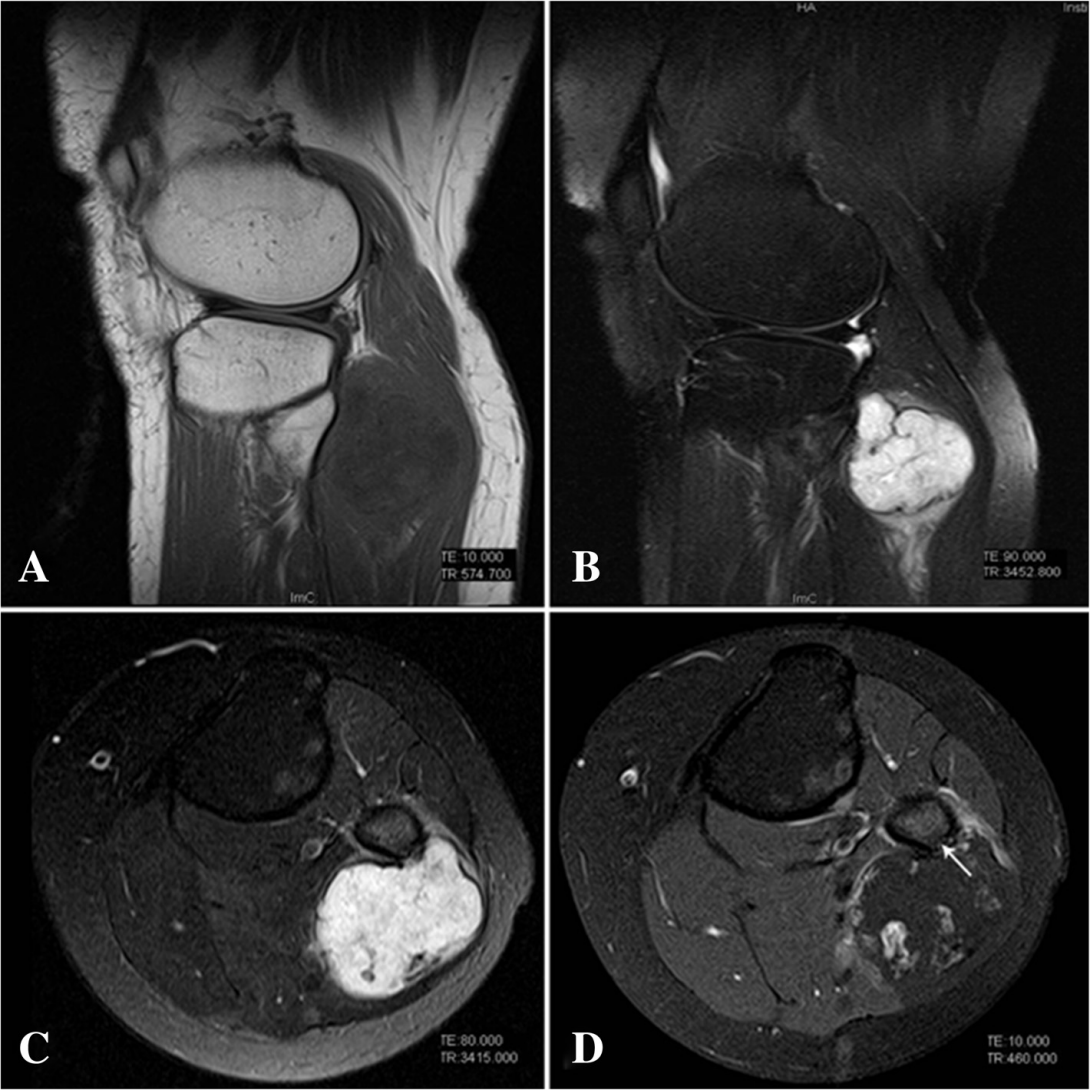
**MRI of tumor. A** Sagittal T1-weight image shows a homogeneous hypo-intensity mass on the posterolateral aspect of the left proximal fibula. **B**, **C** Sagittal and axial T2-weight images show a hyper-intensity mass (5.5 × 5.0 × 4.5 cm) with a multilobular configuration. **D** Axial T1-weighted MRI with fat suppression shows the contrast enhancement around the tumor and cortical invasion (arrow).

**Figure 3:**
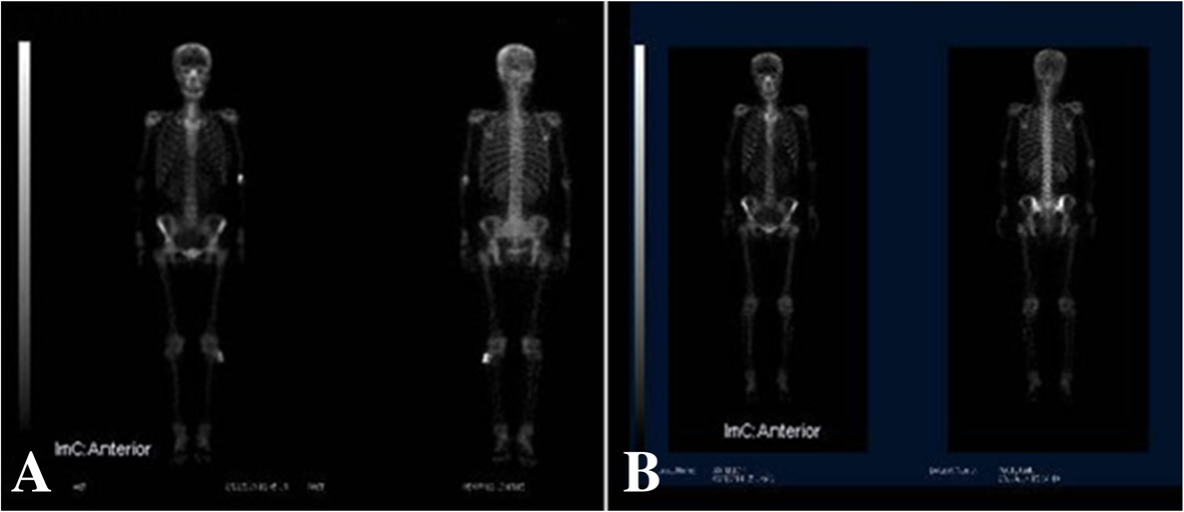
**The whole-body bone scan of the patient.** Bone scan shows strong accumulation of the radiopharmaceutical agent on the outside aspect of the left proximal fibula preoperatively **(A)** and in bilateral iliac regions postoperatively **(B)** without strong accumulation in other parts of the body.

Plain radiograph showed a mass with ‘popcorn’ and ‘smoke ring’ pattern of calcification on the posterolateral aspect of the left proximal fibula. The erosion of underlying cortex was not obvious (Figure 1A, B). A magnetic resonance imaging (MRI) of the knees was done to assess the extent and anatomical position of the mass. MRI showed a vaguely lobulated solid mass measuring 5.5 × 5.0 × 4.5 cm on the posterolateral aspect of the left proximal fibula. The mass exhibited homogeneous low signal intense on T1-weight image (Figure 2A) and high signal intense on T2-weight images (Figure 2B,C). Axial T1-weight MRI with fat suppression showed cortical invasion without marrow involvement (Figure 2D). A whole-body bone scan with technetium-99 m-methylenediphosphonate (Tc-MDP) showed strong accumulation of the radiopharmaceutical agent on the outside aspect of the left proximal fibula, without strong accumulation in other parts of the body (Figure 3A).

Considering the above factors, a primary diagnosis of periosteal malignancy was made, including PC, periosteal osteosarcoma, and parosteal osteosarcoma. Among them, the most likely diagnosis was PC.

A wide resection was performed. The intraoperative result of frozen section examination indicated a PC. According to the pathologic result, an en-bloc resection of the tumor and the left proximal fibula was performed. The thickness of the excised normal soft tissues surrounding the tumor and the length of excised fibula was defined 1.0 and 8.5 cm, respectively. At surgery, the common peroneal nerve was protected carefully and the lateral collateral ligament was reattached to the lateral tibial metaphysis with an anchor.

**Figure 4:**
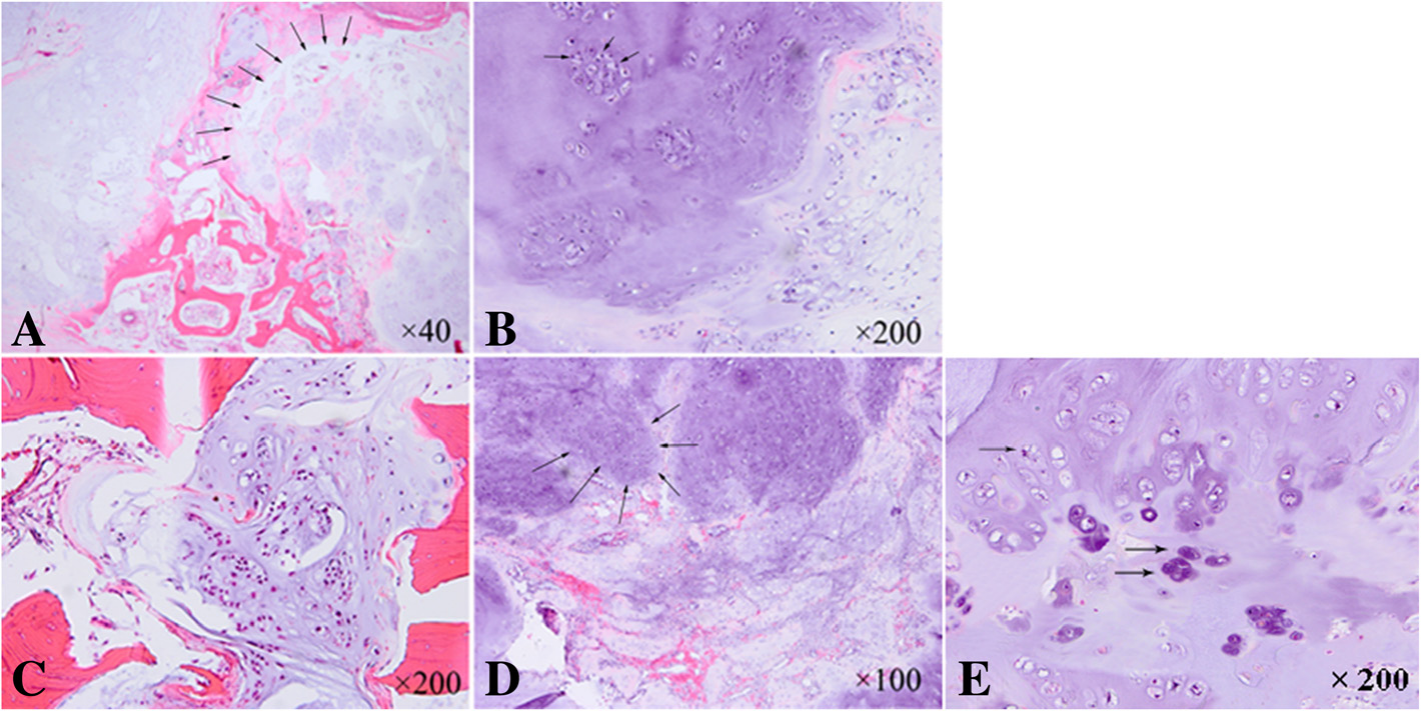
**Histological appearance of tumor (H&E). A** Microscopic appearance of the tissue specimen dissected from the fibula lesion shows a vaguely lobulated neoplastic hyaline cartilage separated by fibrous bands and focal myxoid change (×40). **B** High-power photomicrograph shows the enlarged tumor cells with moderate grade of atypia and exhibits multiple, enlarged grotesque nuclei. Mitoses can be seen (×200). **C** The tumor cells were seen in the Volkman canal (×200). **D**, **E** High-power photomicrograph of the tissue specimen from the needle biopsy of ilium shows a cartilaginous lesion with hypercellularity and cytologic atypia (×100, ×200, respectively).

Postoperative pathological examination confirmed the diagnosis of PC. Hematoxylin and eosin (H&E) staining indicated a vaguely lobulated neoplastic hyaline cartilage separated by fibrous bands and focal myxoid change (Figure 4A). The tumor cells enlarged and presented moderate grade of atypia, including multiple and enlarged nuclei. Mitoses were rare (Figure 4B). At high magnification, the tumor cells were seen in the Volkman canal (Figure 4C). The resection margins were clear. According to the above features and the grading system used for conventional intramedullary chondrosarcomas [1], the histopathological diagnosis of a PC (grade 2) with cortical invasion was rendered.

The patient has been followed up for 16 months. Postoperative recovery was uneventful in the initial follow-up (Figure 1C, D). The patient, who was in a bad physical and mental state, visited our bone tumor clinic with a pain in pelvic region at 10 months after operation. On physical examination, no clinical results were found except the deep tenderness of sacral region and spine. A whole-body bone scan demonstrated high uptake especially in the bilateral iliac regions (Figure 3B).

**Figure 5:**
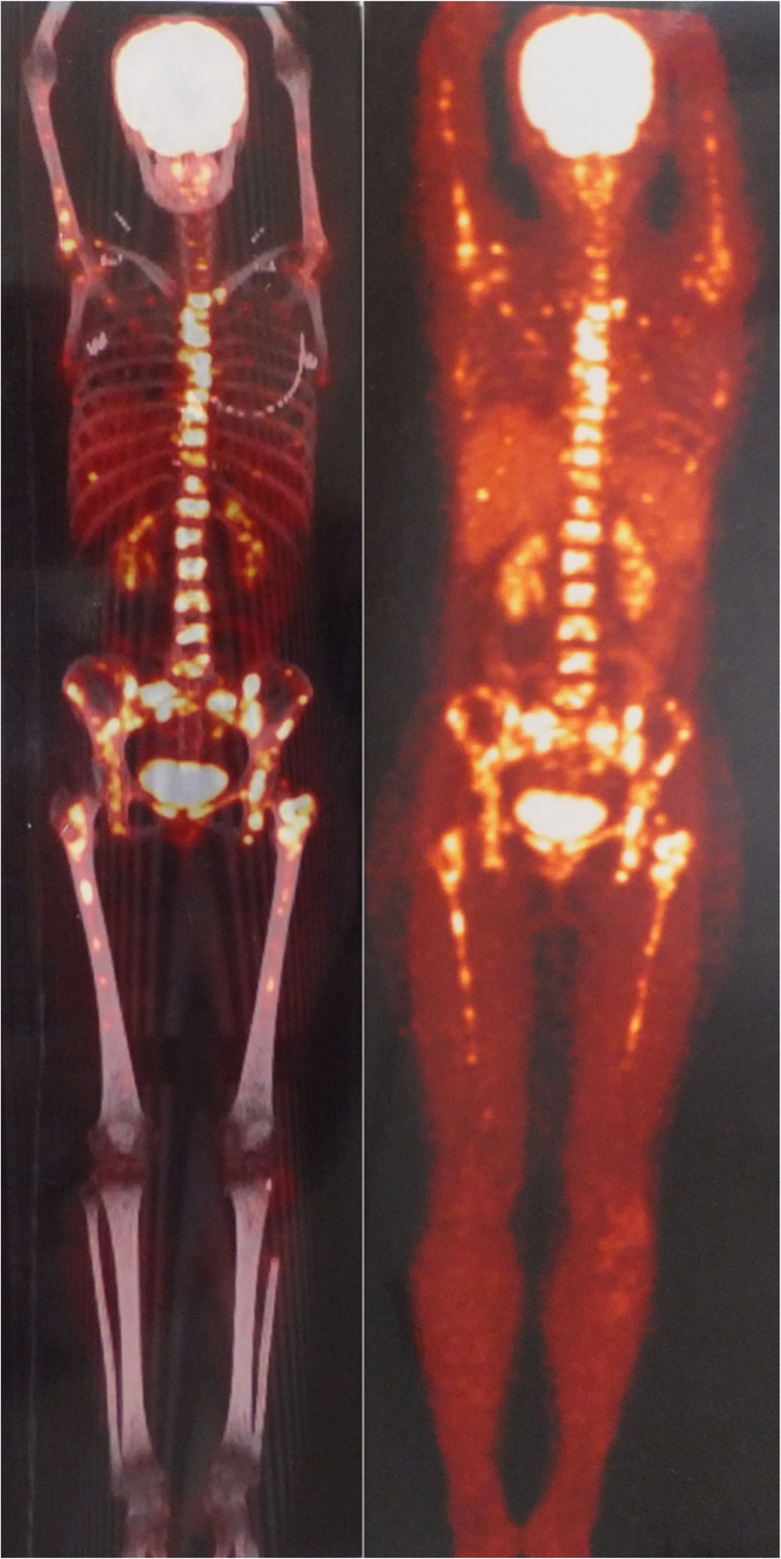
**PET/CT of the patient.** F-18 FDG PET/CT shows multiple bone metastases especially and symmetrically in the axial skeleton and proximal extremities including bilateral femurs and humeri, with a maximum SUV of 10.8, but with no signs of local recurrence and no focal F-18 FDG uptake in the brain, head, neck, chest, and abdomen except a small nodule in the liver which was suspected as a calcification.

In order to further define and assess the extent and anatomical position of metastasis, PET/CT with 2-fluoro [fluorine-18]-2-deoxy-d-glucose (F-18 FDG) was recommended to be done. PET/CT showed focal F-18 FDG uptake especially in the spine, pelvis, bilateral femurs, and humeri, with a maximum SUV of 10.8, but with no signs of local recurrence and no focal F-18 FDG uptake in the brain, head, neck, chest, and abdomen except a small nodule in the liver which was suspected as a calcification (Figure 5). The histopathological result of the needle biopsy of the ilium confirmed the diagnosis of MBM of chondrosarcoma, and the grade of metastatic chondrosarcoma was similar to that of primary PC (Figure 4D, E).

Over the ensuing days, the patient received only the palliative therapy such as analgesic treatment without chemotherapy or radiotherapy. The patient died at 6 months after the definite diagnosis of MBM.

## Discussion

The mean age of PC is about 32 years (range, 20 to 40 years) [2,3,5]. The location mainly exists in the limbs, including the distal femur, proximal tibia, and proximal humerus [2-5]. PC of the fibula is still rare. There have been only two case reports about PC of the fibula. Papagelopoulos *et al.* [6] reported a PC (grade 2) of the left distal fibula of a 31-year-old man measuring 2 cm in size. Robinson *et al.* [7] also reported a PC of the right distal fibula and tibia which induced the underling cortex erosion without marrow invasion of a 64-year-old woman. Lesions of PC are usually 4 to 20 cm in length [2,5]; in most cases, they exceed 5 cm in maximum diameter [5]. Robinson *et al.* [7] concluded that periosteal chondroid lesions greater than 3 cm in length should be considered malignant.

Chemotherapy and radiotherapy are not effective treatments for PC. Wide surgical resection with a sufficient margin remains the recommended therapy for this type of bone tumor. Prognosis is usually good if surgery is adequate [4,8,9]. The reported incidence of local recurrence varies from 13% to 28% and depends on the type of surgical treatment [6,10]. Even when local recurrence occur, the prognosis is still good if the wide resection can be performed again. Lung metastases, even regional lymph node metastases, have been reported in published literatures [6,11,12]. It is important to note that the majority of lung metastases have inadequate surgical treatment of the primary lesion and often accompany with local recurrence. Bone metastasis is even rare. There have been few studies about bone metastasis of chondrosarcoma. Ozaki *et al.* [13,14] reported two cases of chondrosarcoma with bone metastasis and found only five cases of chondrosarcoma with bony metastases by reviewing literatures. They also found all metastases mainly in the axial skeleton or proximal extremities, and there were only three cases of chondrosarcoma with bony metastases without local recurrence. There were also some literatures about bony metastases of extraskeletal myxoid chondrosarcoma and clear cell chondrosarcoma [15-17]. But, there have been no reports about bony metastases of PC.

The mechanism of bony metastases is not very clear. Ozaki *et al.* [14] believed that the metastasis was in the lumbar spine and may not have been associated with lung metastases because of the venous connections between the vertebral and other veins and the absence of a basement membrane in the capillaries of bone marrow. Matsumura *et al.* [18] described that PC microscopically invaded the underlying cortex through the Volkman canal in the cortical bone. Andrew *et al.* [19] reported that although a chondrosarcoma might metastasize late or not at all, it could invade and spread through veins. Death might also result from relentless local growth and invasion of adjacent vital structures. Therefore, considering the above literatures, we deduced that tumor cells had spread through the venules in the Volkman canal before surgery, which could not be tested by the present medical equipments. The microscopic cortical invasion through the Volkman canal and the spread of tumor cells through veins may be the most likely reason for bony metastases without local recurrence in the present case. Other cancers which had not been found before and/or after surgery may be another reason.

In addition, histological grading (grades 1 to 3) for chondrosarcomas is related to clinical behavior and prognosis [9,13]. Grade 1 chondrosarcomas rarely metastasize, while grade 3 chondrosarcmas develop metastases in 70% of patients [9]. Ozaki *et al.* [13] concluded that the median interval between surgery for the primary lesion and metastases was 10 months and the median survival time after metastasis of the patients who could not or did not undergo metastectomy was 5 months (range 0 to 10 months). On the other hand, the survival periods after metastasis of two patients who underwent metastectomy were 15 and 29 months. Papagelopoulos *et al.* [6] also found patients with grade 2 PC had a worse prognosis than patients with grade 1 PC in the 5-year metastasis-free survival (50% versus 94%).

The prognosis of most PC is usually good, but regular follow-up is still important for the early finding of local relapse or metastases. F-18 FDG PET/CT is a recommended examination for finding early metastases. Maybe, the patients with early local metastases will have the opportunity for metastectomy. For the minority of patients with late and multiple metastases, whether chemotherapy is applicable needs further study. New drugs and other treatments are also urgently needed.

## Conclusions

In conclusion, we reported a rare case which has the following features: (1) the location was the proximal fibula, not the common location including distal femur, proximal tibia, and proximal humerus; (2) ‘smoke ring’ pattern of calcification was shown on lateral roentgenogram which has been rarely reported in published literatures; (3) cortical erosion was shown by MRI and histopathological examination which was reported only once; (4) the most important feature is that MBM was shown by F-18 FDG PET/CT especially and symmetrically in the axial skeleton and proximal extremities including bilateral femurs and humeri; and (5) the period from surgery to postoperative metastases and the survival span of our patient was 10 and 6 months, respectively, which was consistent with the previous reports.

Finally, we hope this report will increase the understanding of this rare bone tumor whose prognosis sometimes deviates from the good expectations.

## Consent

Written informed consent was obtained from the patient’s husband for publication of this case report and any accompanying images. The report was approved by the Ethics Committee of our hospital.

## Abbreviations

CT: computed tomography
F-18 FDG: 2-fluoro [fluorine-18]-2-deoxy-d-glucose
H&E: hematoxylin and eosin
MBM: multiple bone metastases
MRI: magnetic resonance imaging
PC: periosteal chondrosarcoma
PET: positron emission tomography
Tc-MDP: technetium-99 m-methylene-diphosphonate
